# Three-Week Treadmill Exercise Enhances Persistent Inward Currents, Facilitates Dendritic Plasticity, and Upregulates the Excitability of Dorsal Raphe Serotonin Neurons in ePet-EYFP Mice

**DOI:** 10.3389/fncel.2020.575626

**Published:** 2020-10-16

**Authors:** Renkai Ge, Yue Dai

**Affiliations:** ^1^Shanghai Key Laboratory of Multidimensional Information Processing, School of Communication and Electronic Engineering, East China Normal University, Shanghai, China; ^2^School of Physical Education and Health Care, East China Jiaotong University, Nanchang, China; ^3^Key Laboratory of Adolescent Health Assessment and Exercise Intervention of Ministry of Education, School of Physical Education and Health Care, East China Normal University, Shanghai, China

**Keywords:** motor control, treadmill exercise, persistent inward currents, dorsal raphe serotonin neurons, excitability

## Abstract

Exercise plays a key role in preventing or treating mental or motor disorders caused by dysfunction of the serotonergic system. However, the electrophysiological and ionic channel mechanisms underlying these effects remain unclear. In this study, we investigated the effects of 3-week treadmill exercise on the electrophysiological and channel properties of dorsal raphe nucleus (DRN). Serotonin (5-HT) neurons in ePet-EYFP mice, using whole-cell patch clamp recording. Treadmill exercise was induced in ePet-EYFP mice of P21–24 for 3 weeks, and whole-cell patch clamp recording was performed on EYFP-positive 5-HT neurons from DRN slices of P42–45 mice. Experiment data showed that 5-HT neurons in the DRN were a heterogeneous population with multiple firing patterns (single firing, phasic firing, and tonic firing). Persistent inward currents (PICs) with multiple patterns were expressed in 5-HT neurons and composed of Cav1.3 (Ca-PIC) and sodium (Na-PIC) components. Exercise hyperpolarized the voltage threshold for action potential (AP) by 3.1 ± 1.0 mV (control: *n* = 14, exercise: *n* = 18, *p* = 0.005) and increased the AP amplitude by 6.7 ± 3.0 mV (*p* = 0.031) and firing frequency by more than 22% especially within a range of current stimulation stronger than 70 pA. A 3-week treadmill exercise was sufficient to hyperpolarize PIC onset by 2.6 ± 1.3 mV (control: −53.4 ± 4.7 mV, *n* = 28; exercise: −56.0 ± 4.7 mV, *n* = 25, *p* = 0.050) and increase the PIC amplitude by 28% (control: 193.6 ± 81.8 pA; exercise: 248.5 ± 105.4 pA, *p* = 0.038). Furthermore, exercise hyperpolarized Na-PIC onset by 3.8 ± 1.8 mV (control: *n* = 8, exercise: *n* = 9, *p* = 0.049) and increased the Ca-PIC amplitude by 23% (*p* = 0.013). The exercise-induced enhancement of the PIC amplitude was mainly mediated by Ca-PIC and hyperpolarization of PIC onset by Na-PIC. Moreover, exercise facilitated dendritic plasticity, which was shown as the increased number of branch points by 1.5 ± 0.5 (*p* = 0.009) and dendritic branches by 2.1 ± 0.6 (*n* = 20, *p* = 0.001) and length by 732.0 ± 100.1 μm (*p* < 0.001) especially within the range of 50–200 μm from the soma. Functional analysis suggested that treadmill exercise enhanced Na-PIC for facilitation of spike initiation and Ca-PIC for regulation of repetitive firing. We concluded that PICs broadly existed in DRN 5-HT neurons and could influence serotonergic neurotransmission in juvenile mice and that 3-week treadmill exercise induced synaptic adaptations, enhanced PICs, and thus upregulated the excitability of the 5-HT neurons.

## Introduction

Serotonin (5-HT) is an essential monoamine synthesized mainly by 5-HT neurons in the dorsal raphe nucleus (DRN). The DRN receives largely synaptic inputs from upstream brain areas (Zhou et al., 2017), constitutes a major source of serotonergic input to the forebrain (Jacobs and Azmitia, 1992), and modulates various functions such as affective, cognitive, rewarding (Liu et al., 2014; Li et al., 2016), and sensory and motor functions (Bacqué-Cazenave et al., 2020). Dysfunction of the serotonergic system has been firmly implicated in depression (Gos et al., 2008; Jacobsen et al., 2012; Mahar et al., 2014) and autism spectrum disorder (Luo et al., 2017). Optogenetic (Warden et al., 2012) or pharmacogenetic (Teissier et al., 2015) activation of serotonin neurons in the DRN leads to antidepressant consequences, which is consistent with the effect of acute selective serotonin reuptake inhibitor (SSRI) administration (Kurt et al., 2000). In addition to mental disorder, dysfunction of the serotonergic system is also closely related to Alzheimer’s disease (Bostanciklioğlu, 2020) and Parkinson’s disease (Fox et al., 2009; Polotis and Niccolini, 2015). Although drugs that target 5-HT receptors and/or 5-HT transporter work well against the above diseases, side effects such as drug resistance and high recurrence rate are also reported in some studies (Mueller et al., 1999). Researchers have been searching for safer and more effective treatments, and they find that exercise can be a promising non-pharmacological treatment strategy for preventing or treating these disorders (Luan et al., 2019). Although the exact underlying mechanism by which exercise improves these conditions is unclear, the effect of exercise on the serotonergic system may be a crucial one. Thirty minutes of acute treadmill exercise at low speed (15 m/min) significantly increased the activation of 5-HT neurons in the DRN and decreased depressive-like behaviors (Otsuka et al., 2016). Acute intensive exercise (2 h at 25 m/min) promotes the synthesis, metabolism, and release of 5-HT (Gomez-Merino et al., 2001). Moreover, a 2-week low intensity treadmill exercise can also increase 5-HT and TPH expressions in the DRN (Ji et al., 2017). Thus, exercise indeed enhances the serotonergic system with the activation of 5-HT neurons no matter whether it is short or long term or low or high intensity. However, the electrophysiological and ionic channel mechanisms underlying this effect remain unclear.

Neurons are remarkable in their capacity to respond and adapt to environmental changes through synaptic plasticity and channel mechanisms. It has been shown in numerous studies that exercise, as one of the environmental changes, improves the excitability of neurons through hyperpolarization of the voltage threshold, increase in action potential (AP) discharge rate, and reduction in rheobase (Beaumont and Gardiner, 2002; Gardiner, 2006; Gardiner et al., 2006; Krutki et al., 2015; Chopek et al., 2018; MacDonell and Gardiner, 2018). Conversely, in the case of restricted activity such as spinal cord transection or suspension of the hindlimbs, changes in the membrane properties are opposite to those induced in the increased activity (Cormery et al., 2005). All these adaptations are due to changes in the number and/or location of channels and receptors. However, the mechanisms by which exercise modulates channels and receptors remain elusive. Persistent inward currents (PICs), long-lasting currents mediated by voltage-gated calcium and sodium channels that inactivate slowly (Powers and Binder, 2003; Harvey et al., 2006; Dai and Jordan, 2011), have been shown to exist in a wide variety of neurons (Schwindt and Crill, 1980; Hounsgaard and Kjaerulff, 1992; Theiss et al., 2007; Dai and Jordan, 2010; Muller et al., 2018; Ge et al., 2019; Revill et al., 2019) and play a crucial role in regulating neuronal excitability (Heckman et al., 2008; Dai et al., 2018). A large number of studies indicate that PICs regulate neuronal excitability through production of plateau potential and bistable firing (Hounsgaard et al., 1984; Hounsgaard and Kiehn, 1993; Lee and Heckman, 1998; Bennett et al., 2001), amplification of synaptic inputs (Lee and Heckman, 2000; Prather et al., 2001; Powers and Heckman, 2017), promotion of firing frequency, and initiation of spike (Harvey et al., 2006; Kuo et al., 2006). As one of the main components of PICs, persistent inward calcium current (Ca-PIC), is mediated by Cav1.3 channels mainly distributed in dendrites (Carlin et al., 2000a,b; Simon et al., 2003) and associated with bistable firing behavior in which a short pulse of excitatory input produces self-sustained firing (Lee and Heckman, 1998; Heckman et al., 2008). Persistent inward sodium current (Na-PIC), another component of PICs, facilitates spike initiation and repetitive firing (Harvey et al., 2006). Previous studies on PICs mainly focused on motoneurons. Recently, we discovered that PICs existed in 5-HT neurons in neonatal mice and played an essential role in regulating the excitability of 5-HT neurons (Ge et al., 2019; Cheng et al., 2020). However, little is known about exercise modulation of PICs in 5-HT neurons. Therefore, we hypothesized that 3-week treadmill exercise modulated the intrinsic and morphological properties of 5-HT neurons in DRN and that these changes in 5-HT neurons could be determined by ionic channel (PIC) modulation, dendritic spasticity, and excitability regulation of the 5-HT neurons.

To address the above issues, we performed a 3-week treadmill exercise in ePet-EYFP mice and explored the effects of exercise on the electrophysiological and channel properties of the 5-HT neurons in the DRN. Our data showed that treadmill exercise significantly increased some measures of excitability of 5-HT neurons in the DRN and enhanced PICs. This study provided insight into the channel mechanism underlying exercise-induced improvement of disorders related to the serotonergic system such as depression.

## Materials and Methods

### Animals and Ethical Approval

The ePet-EYFP mice, expressing enhanced yellow fluorescent protein in 5-HT neurons, were used in this study, which were generated by crossing ePet-cre mice with R26-stop-EYFP mice (Jackson Lab., USA ePet-cre: Stock No: 012712; R26-stop-EYFP: Stock No: 006148; Figure 1A). The number of animals used for this study (total 78 mice) and animal pain and distress were all minimized. Sixty-three mice (P21–24) of either sex were used for electrophysiological recording. These mice were randomly assigned to control (*n* = 30, from six litters) and treadmill exercise (*n* = 33, from seven litters) groups. Since only one or two mice per day could be used for electrophysiological recording, a maximum of six mice per litter were selected to ensure consistent training conditions. Two new mice were added for training each day, and electrophysiological recordings were conducted 24 h after the last exercise. The mice that were finally used for electrophysiological recording were aged at P42–P45. In addition, three normal ePet-EYFP mice at P42 were used for the immunofluorescence experiment to confirm that the transgenic mouse model at this age (P42) was reliable for identifying 5-HT neurons in the DRN. The other 12 mice (P42–45, control: *n* = 6, exercise: *n* = 6) were used for behavior tests. All mice were housed in standard plastic cages (3–6 mice per cage) with a 12-h light/ dark schedule (light: 7 AM to 7 PM) under controlled temperature (22 ± 2°C) and humidity (50 ± 10%). All mice had access to standard laboratory rodent chow and water *ad libitum*. All the experiments were performed in accordance with the ethical requirements of the East China Normal University Public Platform for Innovation and the local Animal Welfare Act and Public Health Service Policy. The protocol involved in this study was approved by the Animal Experiment Ethics Committee (Ethics No. m20190201).

**Figure 1:**
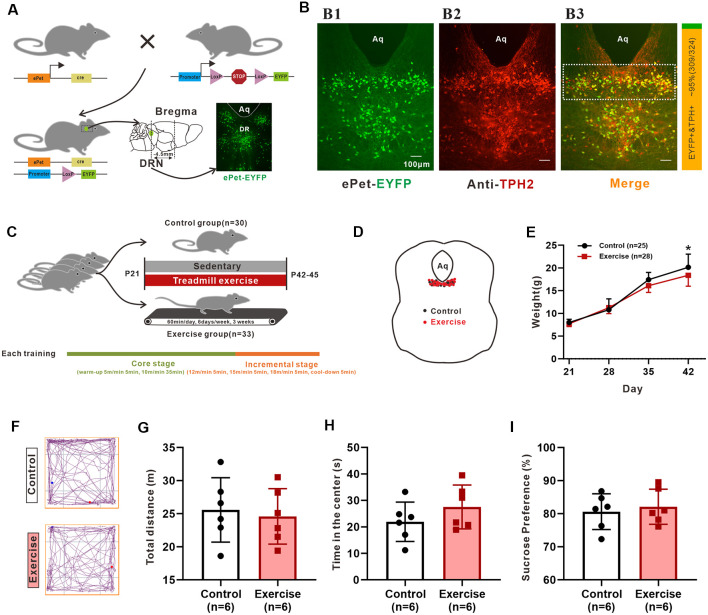
The reliability of animal model and experimental design. **(A)** Schematic representation of the generation of the transgenic ePet-EYFP mice. The Serotonin (5-HT) neurons in the dorsal raphe nucleus (DRN; about −4.5 mm apart from bregma) were labeled with enhanced yellow fluorescent proteins. **(B)** Colocalization **(B3)** of ePet-EYFP **(B1)** and TPH2 (**B2**; TPH2 1:200, ab184505, Abcam) in DRN. The ratio of the number of TPH2-labeled EYFP-positive neurons (orange color, double labeling) to the number of EYFP-positive neurons (both green and orange colors) in the DRN (shown as dotted box in **B3**) were counted manually. The statistical results are shown in a bar chart on the right (about 95%, 309/324, mice: *n* = 3). **(C)** Sixty-three mice (P21) were randomly divided into control group and treadmill exercise group. Control group mice remained sedentary (*n* = 30), and treadmill exercise mice completed 60 min treadmill exercise each day (6 days/week) for a period of 3 weeks (*n* = 33). Each training session was divided into two stages (core stage and incremental stage). The core stage took a total of 40 min (warm-up 5 m/min for 5 min, 10 m/min for 35 min). The incremental stage took a total of 20 min (12 m/min for 5 min, 15 m/min for 5 min, 18 m/min for 5 min, cool-down for 5 min). **(D)** Location of neurons patched for electrophysiological and morphological analysis. **(E)** Weight of control (*n* = 25) and treadmill exercise group (*n* = 28) mice at P21, P28, P35, and P42. Three weeks of exercise significantly decreased the body mass of mice (*p* = 0.036). **(F)** The representative trajectory maps of each group. There was no significant difference either in total distance **(G)** or time in the center **(H)** in the Open-Field Test (OFT; *p* = 0.715, *p* = 0.247, respectively). **(I)** There was no significant difference in sucrose preference (*p* = 0.638). *p < 0.05.

### Treadmill Exercise Protocol

Based on exercise intensity studied in the previous study in mice (Fernando et al., 1993), we designed a moderate-intensity exercise protocol in this study. Three days of acclimatized exercise for 30 min/day at a speed of 5 m/min were performed in the exercise group on a motor-driven treadmill with no incline to allow the mice to familiarize the exercise environment before the formal training. Then, the mice were allowed to run on the treadmill for 60 min/day, 6 day/week, for 3 weeks. Each training session was divided into two stages (core stage and incremental stage). The core stage took a total of 40 min (warm-up at 5 m/min for 5 min, 10 m/min for 35 min), and incremental stage took a total of 20 min (12 m/min for 5 min, 15 m/min for 5 min, 18 m/min for 5 min, cool-down 5 min; Figure 1C). Neither electric shock nor severe physical prodding was used in this study. Only gentle tail touching was used to motivate the mice to run continuously to minimize the stress associated with treadmill exercise. Under these conditions, most of the mice were able to complete the first stage of training well. Incremental stage training is a flexible task, which is mainly for the different individuals’ exercise ability, rather than forcing every mouse to complete it. In this stage, the mice were returned to their cages when they need to be pushed twice or more in a minute to avoid the detrimental stress response caused by increased exercise intensity. During the 3-week exercise training, the general states of the animals such as nutrition, hair brightness, and appetite were monitored. In order to exclude factors that were not induced by the exercise training but may affect the animals such as exposure of the mice to a novel environment, we placed mice from the control group to a stopped treadmill for the same period of experimental time. When the mice were put on the treadmill, they did some exploration activity at the beginning. This exploration activity decreased significantly after the mice became familiar with the environment. In this case, it makes sense to use these mice as control group. The interference of the environment to the result of exercise training is eliminated. Also, this method is widely used in the exercise intervention research, which is susceptible to environmental factors (Li et al., 2013). Except for the exercise intervention, we provided exactly the same conditions for both groups of mice. For both groups, the body mass of mice was measured every week. To reduce the effects of exercise training on the mice’ circadian rhythm, all training sessions took place in the morning (7:00–8:30 a.m.).

### Preparation of Slices

All mice (P42–45) were deeply anesthetized *via* isoflurane and decapitated. The brain was rapidly dissected in the dissecting dish, which was filled with chilled dissecting aCSF and bubbled with carbogen (95% O_2_ and 5% CO_2_). Cerebral cortices and the cerebellum were removed, and only the brainstem was remained. Two hundred fifty-micrometers transverse slices were cut with Leica vibrating microtome (VT 1000E Germany). According to the brain stereogram of the mice plotted by Paxinos and Franklin (2001), the brain slices containing DRN (−4.3 to −4.7 mm caudal to Bregma) were transferred to the recording aCSF for at least 1 h at room temperature before the patch-clamp recordings.

### Whole-Cell Patch-Clamp Recording

The procedure for whole-cell patch-clamp recording was described in previous study (Dai et al., 2009a; Ge et al., 2019). Briefly, the slices were transferred to a recording chamber mounted in the stage of an upright Olympus BX50 microscope fitted with differential interference contrast (DIC) optics and epifluorescence. The chamber was perfused with recording aCSF at a rate of 2 ml/min *via* a gravity-driven irrigation system, bubbled with 95% O_2_ and 5% CO_2_. The EYFP-positive 5-HT neurons in the DRN were identified at 40× magnification using epifluorescence. The visualized neurons within the rectangle area of 1 × 0.3 mm below the cerebral aqueduct that include the dorsomedial and lateral wing subdivisions (Paxinos and Franklin, 2001) and shown as dotted box in Figures 1B,D were patched for electrophysiological and morphological analysis. The pipette electrodes were pulled from borosilicate glass (WPI 1B150F-4) using a VT1000S Sutter puller and had resistances of 5–8 MΩ when filled with intracellular solution. A MultiClamp 700B patch clamp amplifier, Axon Digidata 1550 A/D converter, Minidigi 1B, and pClamp (10.7) software (all from Molecular Devices) were used for data acquisition. Whole-cell patch recordings were made in current-clamp mode with bridge balance and in voltage-clamp mode with capacitance compensation. The liquid junction potential was calculated as 6.6 mV. This value was corrected in the reported data. Data were low filtered at 3 kHz and sampled at 10 kHz.

### Measurement of Membrane Parameters

In this study, current clamp was used to investigate the effects of treadmill exercise on the excitability of 5-HT neurons and the contribution of PICs to neuronal excitability. The main membrane properties measured in current clamp included rheobase (Ith), voltage threshold (Vth), resting membrane potential (Em), input resistance (Rin), AP height and half width, and after-hyperpolarization (AHP) depth, and half-width and number of APs. The calculation of these parameter values had been described in our previous studies (Dai et al., 2009a; Ge et al., 2019; Cheng et al., 2020). Briefly, the rheobase (Ith) was determined by step currents with a 0.5-s duration and 5 pA for each step. The minimum step which evoked a single spike or repetitive firing was defined as rheobase (Ith). The voltage threshold (Vth) was defined as the membrane potential at which the rising rate of dV/dt ≥ 10 mV/ms. The Vth reported in this study was calculated from the spike averaged from the first one to three spikes of firings evoked by the rheobase. The resting membrane potential (Em) was monitored throughout the recordings. The reported Em in this paper was calculated from the membrane potentials averaged over 100 ms prior to the step currents used for determining the rheobase. The measurements of AP and AHP properties were based on the averaged spike evoked by the rheobase. The Vth was used as reference value to measure the AP height and half-width and AHP depth and half-width. The input resistance (Rin) was calculated by the mean value of membrane potentials divided by the amplitude of the corresponding negative step current (0.5 s duration, −5 pA step). In addition, a voltage clamp was used to study the effects of treadmill exercise on PICs in the 5-HT neurons. PICs were elicited by applying five slow voltage ramps from −70 to 90 mV with a 10-s duration and 40-mV peak step. In order to ensure the comparability of the data, the PICs triggered by the fourth ramp (from −70 to +50 mV) were used to calculate PIC parameters in this study. The leak current was subtracted from the recordings before the biophysical parameters of PIC were calculated. The parameters included PIC amplitude, onset voltage, peak voltage, and PIC half-width. Details of the calculation were described in our previous studies (Dai and Jordan, 2010, 2011; Ge et al., 2019). Briefly, only the PICs evoked in the ascending phase of the voltage ramp were used to calculate the parameters. In the case of only one PIC, the onset voltage (Vo) was defined as the voltage value corresponding to the onset of the PIC (Io). The lowest point of the current trough was defined as the peak of PIC (Ip) and the corresponding voltage as peak voltage (Vp). The amplitude of PIC was calculated as the difference between Io and Ip (PIC = Io − Ip). In the case of two PICs, each PIC was calculated in the same way as the case of one PIC. In some neurons, step voltages with step of 10 mV and duration of 1 s were applied to the neurons to generate inward currents, and the normalized current–voltage (I–V) curve was fitted by Boltzmann equation: *f*_(V)_ = 1/{1 + exp[(Vmid − V)/Vc]} to determine the kinetics of the PICs. Clampfit 10.7 was used to analyze the data. All recordings were made at room temperature (20–22°C).

### Immunofluorescence

The mouse (P42) brainstems were fixed with 4% paraformaldehyde at 4°C for 12 h. After fixation, tissues were embedded in low-melting agarose and cut into 30-μm transverse sections with a Leica vibrating microtome (VT 1000E, Germany). Sections were permeabilized with 0.3% Triton X-100 (Sinopharm Chemical Reagent Company Limited) and blocked with 5% bovine serum albumin (BSA, Sinopharm Chemical Reagent Company Limited) in 0.1 M PBS (Sinopharm Chemical Reagent Company Limited) for 30 min. The sections were incubated with primary antibodies: rabbit anti-TPH2 (1:200, ab184505, Abcam) diluted in 5% BSA in PBS at 4°C overnight. Afterward, the sections were washed with 0.1 M PBS (three times, 5 min for each time) and incubated with a goat anti-rabbit Alexa Fluor 594 sary antibody (1:100, A-11012, Invitrogen) diluted in 5% BSA in PBA for 1 h at room temperature. After washing with 0.1 M PBS, the stained neurons were photographed with a fluorescence microscope using 10× and 20× objectives (Nikon Eclipse Ni fluorescence microscopy).

### Imaging and Sholl Analysis

For morphological analysis, part of the EYFP-positive 5-HT neurons were patched and labeled using pipette electrodes filled with intracellular solution containing 3% tetramethylrhodamine. After 5–10 min of staining, images of the labeled neurons were immediately taken by a Nikon Eclipse Ni fluorescence microscopy with a Nikon DS-Ri2 color digital camera at 540–580 and 465–495-nm excitation wavelengths, respectively.

The Sholl analysis was used to quantify neuronal dendritic complexity. It created a series of concentric circles around the focus of a neuronal dendritic arbor and counted how many times connected voxels defining the dendritic arbor intersect the sampling circles as well as the length of each dendritic arbor segment within each radius. In addition, the dendritic branches, dendritic terminals, diameter of soma, and primary dendritic segments were analyzed in the same labeled neuron. The center of concentric spheres was defined as the center of the soma for Sholl analysis, and a 25-μm radius interval was used. The Sholl analysis was obtained using ImageJ software (1.52 g) loaded with Sholl Analysis^1^ and NeuronJ plugins (Meijering et al., 2004)^1^.

### Open-Field Test (OFT)

In order to evaluate the locomotion and anxiety levels of the mice subjected to forced treadmill exercise, an OFT was conducted 24 h after the last exercise session. The OFT was conducted between 7:00 AM and 9:00 PM. The OFT was performed in an unfamiliar plastic box (50 × 50 × 40 cm, length × width × height) with diffuse dim lighting. Mice were released in one corner of the apparatus and allowed to move freely for 5 min. Behaviors were tracked and analyzed by a video tracking software ANY-maze 6.33 (Stoelting Company, USA). The total distance traveled, and the distance traveled in the center area of the apparatus was analyzed.

### Sucrose Preference Test (SPT)

A SPT was used to reflect anhedonia and inversely associated with the severity of depression. SPT was conducted 24 h after the last exercise session. Prior to the test, mice were separated and housed singly in the cages. Two bottles which contained 1% sucrose solution were placed in each cage for the first 24 h. Then, one bottle was replaced by tap water for the next 24 h. To prevent possible effects of side preference in drinking behavior, the position of the bottles was switched after 12 h. After 12 h of food and water deprivation, mice were tested by freely accessing two bottles containing either 1% sucrose solution or tap water for 24 h. Water and sucrose consumption was measured by evaluating changes in the weight of fluid consumed. Sucrose preference (%) was calculated based on the formula: sucrose preference (%) = sucrose intake (g)/[sucrose intake (g) + tap water intake (g)] × 100%.

### Solutions and Chemicals

Dissecting artificial cerebrospinal fluid (aCSF) contained (in mM): NaCl (25), sucrose (188), KCl (1.9), NaH_2_PO_4_ (1.2), MgSO_4_ (10), NaHCO_3_ (26), kynurenic acid (1.5), glucose (25), and CaCl_2_ (1.0).

Recording aCSF containing (in mM): NaCl (125), KCl (2.5), NaHCO_3_ (26), NaH_2_PO_4_ (1.25), glucose (25), MgCl_2_ (1), and CaCl_2_ (2.0), TEA (10, only in voltage clamp mode).

Intracellular solution containing (in mM): K-gluconate (130), NaCl (10), HEPES (10), MgCl_2_ (2), Mg-ATP (5), and GTP (0.5), TEA-Cl (20, only in voltage clamp mode).

The pH of these solutions was adjusted to 7.3 with HCl. Osmolarity was adjusted to 305 mOsm by adding sucrose to the solution.

Drugs: Stock solutions of all drugs (10 mM) were prepared in DMSO and stored at −20°C. All drugs were dissolved in normal aCSF at a final concentration and superfused in the recording chamber at 2 ml/min from a 5-channel gravity-driven perfusion system for 10 min in each condition. DMSO in the final concentration of experimental drugs in the bath was kept under 0.02%. Two micrometer TTX (HY-12526; MCE) was used to block the TTX-sensitive transient and persistent inward sodium currents. Two micrometer riluzole (HY-B0211; MCE) was used to block the persistent inward sodium current. Twenty-five micrometer nimodipine (HY-B0265; MCE) was used to block the DHP-sensitive L-type calcium PIC.

### Statistical Analysis

Microsoft Excel (Office 2019) was used for data formatting, and statistical analysis was carried out using IBM SPSS statistics (version 22). The data were presented in means ± SD. Normal distribution of experimental data was assessed through the Shapiro–Wilk test. Data were analyzed using unpaired Student’s *t*-test, chi-square tests, and repeated-measures two-way ANOVA. The Newman–Keuls *post hoc* test was performed when appropriate. The nonparametric Mann–Whitney’s *U* test was used for those data which were not normally distributed. In all cases, *p* < 0.05 was considered statistically significant.

## Results

### The Reliability of Animal Model and Experimental Design

Pet-1, an ETS-domain transcription factor, is a precise marker of 5-HT neurons (Scott et al., 2005). In the present study, ePet-EYFP mice were generated by crossing ePet-cre mice with R26-stop-EYFP mice, in which the 5-HT neurons were labeling an enhanced yellow fluorescent protein (EYFP; Figure 1A). The live 5-HT neurons could be accurately identified and recorded. We relied on the use of Cre recombinase to faithfully express eYFP in post-mitotic 5-HT-producing neurons. However, as with any genetic lines, embryonic expression may produce fluorescent positive post-mitotic cells that no longer express the gene/protein of interest. To confirm that these EYFP-positive neurons in the DRN of 42-day mice were 5-HT-positive neurons, three mice (P42) were used to perform an immunofluorescence experiment. One example is shown in Figure 1B, where most EYFP-positive neurons (Figure 1B) were labeled by TPH2 (Figure 1B). TPH2-labeled EYFP-positive neurons within the rectangle area of 1 × 0.3 mm below the cerebral aqueduct in the DRN were counted manually from the merged pictures in Figure 1B (showed as dotted box). Statistical results showed that approximately 95% (309/324, mice: *n* = 3) of EYFP+ neurons expressed TPH2 (Figure 1B, right), which was similar to the previous study (Pinto et al., 2019). Thus, the mouse model at this age was reliable for studying 5-HT neurons in the DRN.

Repeated-measures ANOVA analyses detected significant main effects of training (*F*_(1,51)_ = 4.746, *p* = 0.034), time (*F*_(3,49)_ = 1883.1, *p* < 0.001), and training × time interaction (*F*_(3,49)_ = 10.03, *p* < 0.001) on weight. As shown in Figure 1E, a 3-week treadmill exercise resulted in decreased weight by 1.5 ± 0.7 g (*p* = 0.036). Conflicting effects of treadmill exercise on emotional behaviors and neuronal plasticity are seen in some studies (Burghardt et al., 2004; Svensson et al., 2016). These discrepancies are related to a number of factors, such as animal model, forced or voluntary exercise protocols, the intensity and duration of exercise, and different individuals’ exercise ability. Inappropriate exercise programs can cause anxiety and even depression in animals. In this study, behavior tests were performed to confirm reliability of experimental design. OFT results showed that there was no significant difference in total distance (Figure 1G, *p* = 0.715) and time in the center (Figure 1H, *p* = 0.247) between control and exercise groups. The representative trajectory maps of each group are shown in Figure 1F. On the other hand, the results from sucrose preference experiments showed that there was no significant difference between two groups (Figure 1I, *p* = 0.638). In addition, the general state of the mice such as nutrition, hair brightness, and appetite were all monitored during exercise experiment, and all mice were kept in a good state. These results suggested that the protocol used for the 3-week treadmill training in this study did not induce anxious or depressed behavior in juvenile mice.

### Multiple Firing Patterns of 5-HT Neurons in the DRN

**Figure 2:**
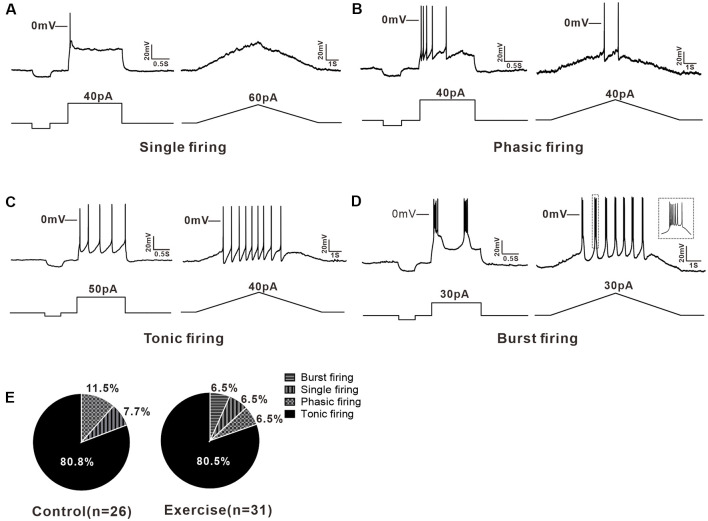
Multiple firing patterns of 5-HT neurons in the DRN. **(A)** A typical example of single firing neuron, the injected currents (step or ramp) elicited only one action potential (AP). **(B)** Step current evoked a brief firing in phasic firing type neuron. **(C)** The tonic firing was evoked by the injected currents (step or ramp) in a tonic firing type neuron. **(D)** Burst firing, a special tonic firing, was elicited by the injected step or ramp currents. **(E)** The proportion of each type of neuron in the control and exercise group. A chi-square test showed no significant group difference in proportion of firing patterns (χ^2^ = 0.711, *df* = 2, *p* = 0.866).

A total of 57 neurons (control: *n* = 26 from 13 mice; exercise: *n* = 31 from 15 mice) were recorded for current clamp analysis. 5-HT neurons in the DRN could be divided into three types based on their firing patterns in response to depolarizing currents. 7.7% (2/26) of neurons in the control group and 6.5% (2/31) of neurons in the exercise group were elicited only one or two APs when depolarizing current injected, and these neurons were classified as single firing type (Figure 2A). 11.5% (3/26) of neurons in the control group and 6.5% (2/31) of neurons in the exercise group discharged briefly at the beginning of the current injection and exhibited a spike frequency adaptation. This type of neurons was classified as phasic firing type (Figure 2B). Most of the 5-HT neurons, 80.8% (21/26) in the control group and 80.5% (25/31) in the exercise group, fired repetitively over the entire current injection and were classified as tonic firing type (Figure 2C). In addition, 6.5% (2/31) of 5-HT neurons in the exercise group displayed a burst pattern of firing (Figure 2D), and this type could be classified as tonic firing type. A chi-square test showed no significant group difference in proportion of firing patterns (χ^2^ = 0.711, *df* = 2, *p* = 0.866; Figure 2E). These results demonstrated that 5-HT neurons in the DRN were heterogeneous, and firing patterns were not obviously affected by exercise training.

### Treadmill Exercise Increased Some Measures of Excitability of 5-HT Neurons

**Figure 3:**
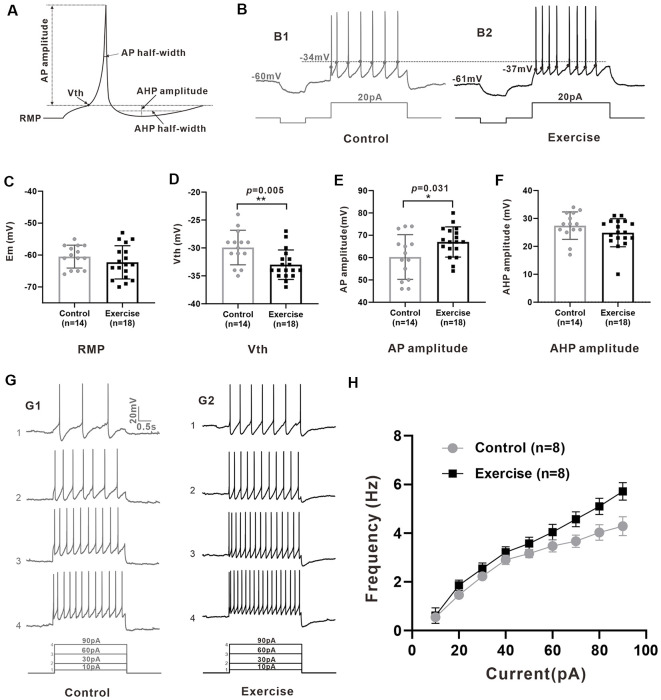
Treadmill exercise increased some measures of excitability of 5-HT neurons. **(A)** Schematic of calculation of neuron active and passive membrane properties. **(B)** The voltage threshold of neurons in exercise training group (**B2**, −37 mV) was hyperpolarized compared with that in the control group (**B1**, −34 mV). **(C)** The resting membrane potential (Em) showed a tendency of hyperpolarization after exercise (*p* = 0.285). **(D)** The voltage threshold (Vth) of 5-HT neurons in the exercise group was significantly hyperpolarized than that of the control group (*p* = 0.005). **(E)** The AP amplitude of 5-HT neurons in the exercise group was bigger than that of the control group (*p* = 0.031). **(F)** No significant difference was found in after-hyperpolarization (AHP) amplitude (*p* = 0.270). **(G)** A typical example showed that under the same current input (bottom), the AP frequency of neurons in the exercise group **(G1)** was significantly higher than that in the control group **(G2)**. **(H)** Two-way ANOVA with repeated measures detected a significant main effect of injected currents, with frequency increasing (*F*_(8,5)_ = 25.317, *p* = 0.001). There was no significant effect of exercise training (*F*_(1,12)_ = 4.196, *p* = 0.063) or current × training interaction (*F*_(8,5)_ = 4.361, *p* = 0.061). Mean ± SEM. *p < 0.05, **p < 0.01.

Measurement of membrane properties was shown in Figure 3A. A typical example is shown in Figure 3B, where Vth was hyperpolarized by 3 mV from −34 to −37 mV with a 3-week treadmill exercise (Figures 3B1,B2). Statistical results showed that the Vth of 5-HT neurons in the treadmill exercise group was hyperpolarized by 3.1 ± 1.0 mV than that in the control group (Figure 3D, control: *n* = 14 from six mice, exercise: *n* = 18 from eight mice, *p* = 0.005). At the same time, the AP amplitude increased 6.7 ± 3.0 mV in the treadmill exercise group (Figure 3E, control: *n* = 14 from six mice, exercise: *n* = 18 from eight mice, *p* = 0.031) whereas no substantial change was observed in resting membrane potential (Figure 3C, control: *n* = 14 from six mice, exercise: *n* = 18 from eight mice, *p* = 0.285), AHP amplitude (Figure 3F, control: *n* = 14 from six mice, exercise: *n* = 18 from eight mice, *p* = 0.27), and rheobase (control: *n* = 14 from six mice, exercise: *n* = 18 from eight mice, *p* = 0.612) in the exercise group. The details of these results are shown in Table 1. Furthermore, there was a tendency that exercise training increased the firing frequency of 5-HT neurons. An example is shown in Figure 3G, where the firing frequency of 5-HT neurons appeared to be higher in the treadmill exercise group (Figure 3G) than in the control group under the same current injection (Figure 3G). Two-way ANOVA with repeated measures detected a significant main effect of injected currents, with frequency increasing (*F*_(8,5)_ = 25.317, *p* = 0.001). There was no significant effect of exercise training (*F*_(1,12)_ = 4.196, *p* = 0.063) or current × training interaction (*F*_(8,5)_ = 4.361, *p* = 0.061; Figure 3H). The above results suggested that treadmill exercise significantly increased some measures of excitability of 5-HT neurons in the DRN. In the following experiments, we would show that this increase in neuronal excitability could be produced by modulating ionic channels.

**Table 1:** Exercise effects on membrane properties of Serotonin (5-HT) neurons.

	Control (*n* = 14)	Exercise (*n* = 18)	Change	*p*-values
Em, mV	−60.5 ± 3.6	−62.3 ± 5.2	−1.8 ± 1.6	0.285
Rin, MΩ	1,050.4 ± 300.0	1,009.4 ± 179.4	−41.0 ± 80.9	0.616
Vth, mV	−29.9 ± 3.1	−33.0 ± 2.6	−3.1 ± 1.0**	0.005
Rheobase, pA	21.1 ± 9.4	19.8 ± 5.9	−1.3 ± 2.5	0.612
AP amplitude, mV	60.3 ± 10.0	67.0 ± 6.8	6.7 ± 3.0*	0.031
AP half-width, ms	2.6 ± 0.8	2.4 ± 0.7	−0.2 ± 0.2	0.450
AHP amplitude, mV	27.4 ± 4.9	25.8 ± 3.4	−1.6 ± 1.5	0.270
AHP 1/2 decay, ms	445.8 ± 111.9	406.6 ± 119.4	−39.2 ± 43.9	0.379

*Significant difference with *p* < 0.05; **significant difference with *p* < 0.01.

### Treadmill Exercise Enhanced the PICs

**Figure 4:**
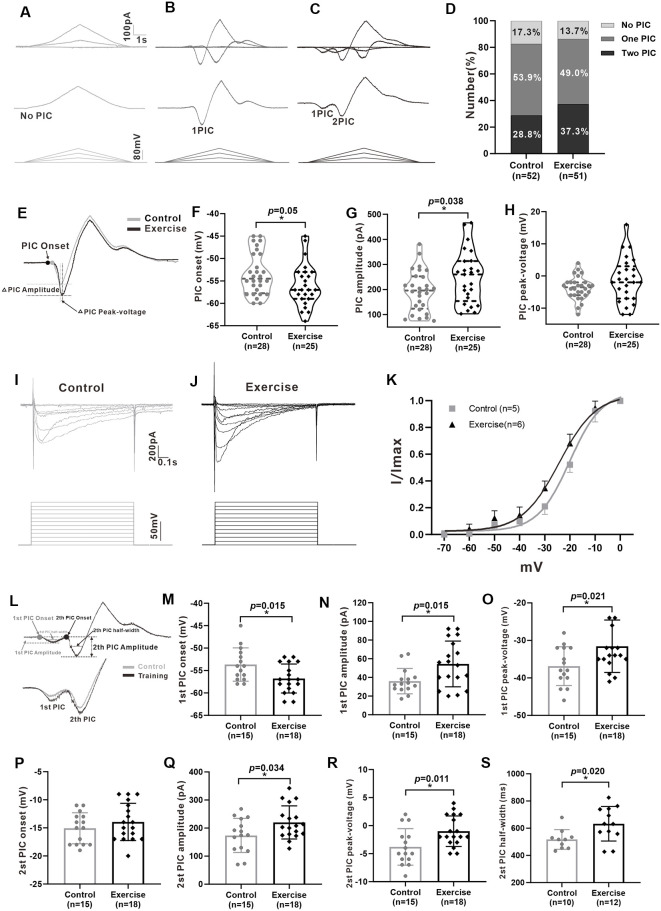
Treadmill exercise enhanced the persistent inward currents (PICs). **(A–C)** Examples of different patterns of PICs (**A**: no PIC; **B**: one PIC; **C**: two PICs) in 5-HT neurons. **(D)** The percentage of different PIC pattern neurons in the control group and the exercise group. **(E)** Examples of treadmill exercise changed parameters of PICs in neurons with only one PIC. **(F)** Treadmill exercise hyperpolarized the PIC onset in neurons with only one PIC (*p* = 0.050). **(G)** Treadmill exercise significantly increased the PIC amplitude in neurons with only one PIC (*p* = 0.038). **(H)** There was no significant change in PIC peak-voltage between two groups (*p* = 0.235). Examples of PICs elicited by a succession of voltage steps with a step of 10 mV (bottom traces) in the control **(I)** and exercise groups. **(J)** PIC traces were used to establish a normalized current–voltage curve, which was then fitted by the Boltzmann function. The kinetic parameters half-maximal activation (Vmid) and activation rate (Vc) were determined for the PICs. Statistical results were shown for voltage dependency of PICs in the control and exercise groups **(K)**. **(L)** Measurement of PIC parameters in neurons with two PICs (up) and an example of the effects of treadmill exercise on PICs (bottom). Statistical results showed that treadmill exercise significantly hyperpolarized the onset (**M**, *p* = 0.015) and increased the amplitude (**N**, *p* = 0.015) and peak-voltage (**O**, *p* = 0.021) of the first PIC. **(P)** There was no significant change in PIC onset of the second PIC between two groups. Statistical results demonstrated that treadmill exercise significantly increased the amplitude (**Q**, *p* = 0.034), peak-voltage (**R**, *p* = 0.011), and PIC half-width (**S**, *p* = 0.020) of the second PIC. *p < 0.05.

Previous studies have shown that PICs play an important role in regulating neuronal excitability and firing patterns in spinal motoneurons and interneurons (Theiss et al., 2007; Heckman et al., 2008). Recently, we found the presence of PICs in 5-HT neurons in the mesencephalic locomotor region and medulla of neonatal ePet-EYFP mice (Ge et al., 2019; Cheng et al., 2020). In this study, we further explored whether PICs presented in 5-HT neurons of DRN in juvenile mice as well as the effects of exercise training on PICs. A total of 103 neurons (control: *n* = 52 from 17 mice; exercise: *n* = 51 from 18 mice) were recorded for PIC analysis. Results showed that PICs were widely expressed in the 5-HT neurons of DRN both in the control group (82.7%, 43/52) and in the treadmill exercise group (86.3%, 44/51). 17.3% (9/52) of the neurons in the control group and 13.7% (7/51) of the neurons in the treadmill exercise group did not express PICs (Figures 4A,D). 53.9% (28/52) of the neurons in the control group and 49% (25/51) of the neurons in the treadmill exercise group displayed one PIC (Figures 4B,D). 28.8% (15/52) of the neurons in the control group and 37.3% (19/51) of the neurons in the treadmill exercise group expressed two PICs (Figures 4C,D). A chi-square test showed no significant group difference in proportion of PIC type (χ^2^ = 0.881, *df* = 2, *p* = 0.644). Calculation of PICs parameters was shown in Figures 4E,L. The 3-week treadmill exercise hyperpolarized PIC onset by 2.6 ± 1.3 mV from −53.4 ± 4.7 to −56.0 ± 4.7 mV (Figure 4F, *p* = 0.050) and increased PIC amplitude by 54.9 ± 25.8 pA from 193.6 ± 81.8 to 248.5 ± 105.4 pA (Figure 4G, *p* = 0.038) in the neurons with one PIC. There was no significant difference in peak-voltage between the two groups (Figure 4H). Similarly, exercise also significantly hyperpolarized *Vmid* by 4.1 ± 1.1 mV from −19.9 ± 2.0 to −24 ± 1.7 mV (Figure 4K, *p* = 0.004) and increased *Vc* by 0.9 ± 1 mV from 6.3 ± 0.6 to 7.2 ± 2.2 mV (Figure 4K, *p* = 0.362). Typical examples were shown in Figures 4I,J. In addition, the 3-week treadmill exercise hyperpolarized the onset of the first PIC by 3.1 ± 1.2 mV from −53.7 ± 3.7 to −56.8 ± 3.2 mV (Figure 4M, *p* = 0.015) and did not affect second PIC onset (Figure 4P). In the neurons of two PICs, treadmill exercise increased the first and second PIC amplitudes by 18.4 ± 7.1 pA (from 36.1 ± 13.6 to 54.4 ± 24.5 pA, Figure 4N, *p* = 0.015) and 46.5 ± 20.9 pA (from 173.6 ± 60.8 to 220.1 ± 59.0 pA, Figure 4Q, *p* = 0.034), respectively, and increased the first and second PIC peak voltages by 5.3 ± 2.2 mV (from −36.9 ± 5.2 to −31.6 ± 7.0 mV, Figure 4O, *p* = 0.021) and 2.8 ± 1.0 mV (from −3.8 ± 3.2 to −1.0 ± 2.7 mV, Figure 4R, *p* = 0.011), respectively. The half-width of the second PIC in the treadmill group significantly increased by 114.9 ± 45.3 ms, from 517.5 ± 72.2 to 632.4 ± 126.9 ms (Figure 4S, *p* = 0.020). These results demonstrated that PICs broadly existed in 5-HT neurons of DRN in juvenile mice and the 3-week treadmill exercise increased the amplitude and hyperpolarized the onset of PICs in 5-HT neurons of DRN.

### Effects of Treadmill Exercise on Multiple Components of PICs

**Figure 5:**
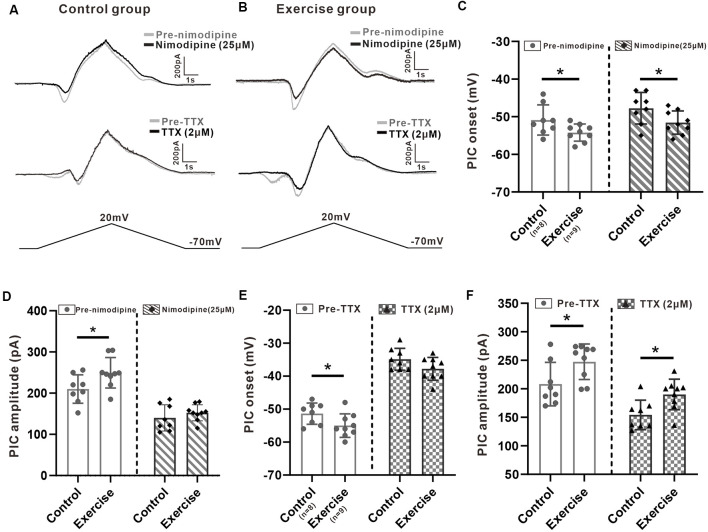
Effects of treadmill exercise on each component of PICs. **(A)** Examples of the effects of nimodipine (25 μM, top traces) and TTX (2 μM, middle traces) on PICs in the control group. **(B)** Examples of the effects of nimodipine (25 μM, top traces) and TTX (2 μM, middle traces) on PICs in the exercise group. **(C)** Exercise training significantly hyperpolarized the onset of composite PIC (left panel, unpaired Student’s *t*-test, *p* = 0.049). Significant difference was also observed in Na-PIC onset between control and exercise groups with nimodipine (right panel, unpaired Student’s *t*-test, *p* = 0.049). **(D)** Exercise training significantly increased the composite PIC amplitude (left panel, unpaired Student’s *t*-test, *p* = 0.039). There was no significant difference in Na-PIC amplitude between the control and exercise groups (right panel, unpaired Student’s *t*-test, *p* = 0.345). **(E)** Exercise training significantly hyperpolarized the onset of composite PIC (left panel, unpaired Student’s *t*-test, composite *p* = 0.046). There was no significant difference in Ca-PIC onset between the control and exercise groups (right panel, unpaired Student’s *t*-test, *p* = 0.098). **(F)** Exercise training significantly increased the amplitude of composite PIC (left panel, unpaired Student’s *t*-test, *p* = 0.034). However, even reduced by TTX, the amplitude of Ca-PIC measured from the exercise group was still bigger than that from the control group (right panel, unpaired Student’s *t*-test, *p* = 0.013). *p < 0.05.

It has been shown in our recent study that PICs in 5-HT neurons of the medulla displayed multiple components including Na-PIC and Ca-PIC. In this study, we investigated the effects of exercise on these two types of PICs. The composite PIC was recorded first and then Na-PIC or Ca-PIC was recorded with bath application of nimodipine (25 μM) or TTX (2 μM), respectively. In this case, both composite PIC and Na-PIC or Ca-PIC were recorded from the same neuron. A typical example showed that 25 μM nimodipine obviously reduced PIC amplitudes with little change in PIC onset in the control and exercise groups (Figures 5A,B, top traces). Two micrometer TTX significantly depolarized PIC onset and partly decreased PIC amplitudes in the control and exercise groups (Figures 5A,B, middle traces). These results suggested that in general, Ca-PIC determined the amplitude of composite PIC and Na-PIC dominated the onset of composite PIC in both control and exercise groups. Na-PIC also determined the amplitude of the first PIC for the staircase PICs (Figure 5B).

First, we investigated the effect of exercise training on Na-PIC with bath application of nimodipine. The experiment data showed that exercise training hyperpolarized the onset of composite PIC by 6.4% from −50.9 ± 4 to −54.2 ± 2.3 mV (Figure 5C, left panel, unpaired Student’s *t*-test, *p* = 0.049, control: *n* = 8 from four mice; exercise: *n* = 9 from five mice). Exercise training also hyperpolarized the onset of Na-PIC by 6.7% from −47.8 ± 4.2 to −51.6 ± 3.1 mV (Figure 5C, right panel, unpaired Student’s *t*-test, *p* = 0.049, control: *n* = 8 from four mice; exercise: *n* = 9 from five mice). These results suggested that the exercise-induced hyperpolarization of composite PIC onset could be mainly mediated by Na-PIC.

Further experiment results showed that exercise training increased the composite PIC amplitude by 18.6% from 201 ± 34.6 to 249.4 ± 37 pA (Figure 5D, left panel, unpaired Student’s *t*-test, *p* = 0.039, control: *n* = 8 from four mice; exercise: *n* = 9 from five mice). However, there was no significant difference in Na-PIC amplitude between control and exercise groups in the presence of nimodipine (Figure 5D, right panel, unpaired Student’s *t*-test, *p* = 0.345, control: *n* = 8 from four mice; exercise: *n* = 9 from five mice). Therefore, these results suggested that the exercise-induced enhancement of PIC amplitude could be primarily determined by Ca-PIC rather than Na-PIC.

We further explored the effect of exercise training on Ca-PIC with bath application of TTX. Our data showed that exercise training significantly hyperpolarized the onset of composite PIC by 3.6 ± 1.7 mV, from −51.4 ± 3.2 to −55 ± 3.6 mV (Figure 5E, left panel, unpaired Student’s *t*-test, *p* = 0.046, control: *n* = 8 from five mice; exercise: *n* = 9 from six mice). However, there was no significant difference in Ca-PIC onset between control and exercise groups (Figure 5E, right panel, unpaired Student’s *t*-test, *p* = 0.098, control: *n* = 8 from five mice; exercise: *n* = 9 from six mice), suggesting again that the exercise-induced hyperpolarization of PIC onset could be mainly mediated by Na-PIC rather than Ca-PIC.

In addition to PIC onset, exercise training also enhanced the composite PIC amplitude by 18.7%, from 208.2 ± 38.1 to 247.4 ± 31.1 pA (Figure 5F, left panel, unpaired Student’s *t*-test, *p* = 0.034, control: *n* = 8 from five mice; exercise: *n* = 9 from six mice). The amplitude of Ca-PIC measured from the exercise group was larger than that from the control group (Figure 5F, right panel, unpaired Student’s *t*-test, *p* = 0.013, control: *n* = 8 from five mice; exercise: *n* = 9 from six mice), suggesting again that the exercise-induced enhancement of PIC amplitude could be dominated by Ca-PIC.

The above results suggested that the exercised-induced hyperpolarization of the PIC onset could be mainly determined by Na-PIC while the exercised-induced enhancement of the PIC amplitude could be dominated by Ca-PIC.

### Effects of PICs on Neuronal Excitability

**Figure 6:**
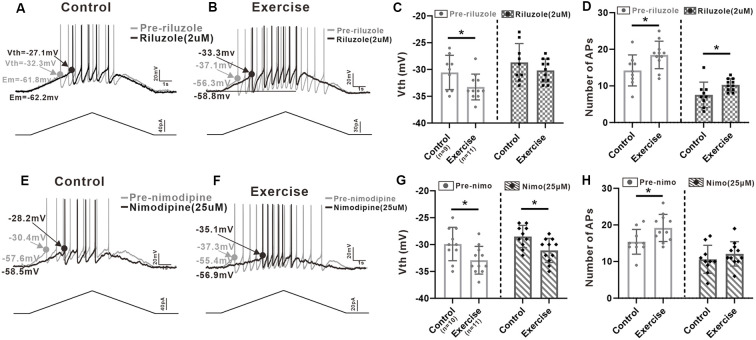
Effects of PICs on neuronal excitability. **(A)** Example of riluzole (2 μM, black trace) reduced the firing frequency of 5-HT neuron compared to that of pre-riluzole (gray trace) and depolarized the Vth in control group. **(B)** Example of riluzole (2 μM, black trace) reduced the firing frequency of 5-HT neuron compared to that of pre-riluzole (gray trace) and depolarized the Vth in the exercise group. **(C)** Exercise training significantly depolarized the Vth (left panel, unpaired Student’s *t*-test, *p* = 0.044). After administration of riluzole, there was no significant difference in Vth between the control and exercise groups (right panel, unpaired Student’s *t*-test, *p* = 0.248). **(D)** Compared with the control group, exercise training increased the number of APs (left panel, unpaired Student’s *t*-test, *p* = 0.029). Even reduced by riluzole, the number of AP measured from the exercise group were still bigger than those from the control group (right panel, unpaired Student’s *t*-test, *p* = 0.034). **(E)** Example of nimodipine (25 μM) reduced the firing frequency of 5-HT neuron compared to that of pre-nimodipine (gray trace). **(F)** Example of nimodipine (25 μM) reduced the firing frequency of 5-HT neuron compared to that of pre-nimodipine (gray trace) in the exercise group. **(G)** Exercise training significantly hyperpolarized the Vth (left panel, unpaired Student’s *t*-test, *p* = 0.026). Significant difference was also observed in Vth between control and exercise groups with nimodipine (right panel, unpaired Student’s *t*-test, *p* = 0.013). **(H)** Compared with the control group, exercise training increased the number of APs (left panel, unpaired Student’s *t*-test, *p* = 0.025). After administration of nimodipine, there was no significant difference in the number of APs between control and exercise groups (right panel, unpaired Student’s *t*-test, *p* = 0.352). *p < 0.05.

In order to investigate contribution of PICs to regulation of neuronal excitability, we injected a slow bi-ramp current with duration of 10 s, peak of 60 pA, and holding current of 0 pA into the 5-HT neurons. This current protocol could generate slow depolarization and hyperpolarization of membrane potential similar to those produced by our voltage protocol for measurement of PICs. The voltage threshold (Vth) of the first spike and the number of spikes of the repetitive firings were calculated (Figures 6A,B,E,F).

Results showed that exercise training hyperpolarized the Vth by 2.7 ± 1.2 mV from −30.6 ± 3.2 to −33.3 ± 2.4 mV (Figure 6C, left panel, unpaired Student’s *t*-test, *p* = 0.044, control: *n* = 9 from five mice; exercise: *n* = 11 from six mice). Although the Vth recorded with riluzole in the exercise group was still lower than the Vth in control, there was no significant difference in Vth between control and exercise groups (Figure 6C, right panel, unpaired Student’s *t*-test, *p* = 0.248, control: *n* = 9 from five mice; exercise: *n* = 11 from six mice), suggesting that exercise training might modulate Na-PIC for spike initiation.

We also compared the number of AP recorded with riluzole. Experiment data showed that exercise training increased the AP numbers by 4.2 ± 1.8 from 14.2 ± 4.2 to 18.4 ± 3.7 (Figure 6D, left panel, unpaired Student’s *t*-test, *p* = 0.029, control: *n* = 9 from five mice; exercise: *n* = 11 from six mice). However, even reduced by riluzole, the number of AP measured from the exercise group was still bigger than that from the control group, which was putative to be regulated by Ca-PIC (Figure 6D, right panel, unpaired Student’s *t*-test, *p* = 0.034, control: *n* = 9 from five mice; exercise: *n* = 11 from six mice), suggesting that exercise training potentially enhanced Ca-PIC for regulation of repetitive firing.

In this study, we also explored the Na-PIC property with nimodipine. Our data showed that exercise training hyperpolarized the Vth by 3 ± 1.2 mV from −29.9 ± 3.1 to −32.9 ± 2.6 mV (Figure 6G, left panel, unpaired Student’s *t*-test, *p* = 0.026, control: *n* = 10 from five mice; exercise: *n* = 11 from six mice). Significant difference was also observed in Vth between control and exercise groups with nimodipine (−2.6 ± 0.9 mV, Figure 6G, right panel, control: *n* = 10 from five mice; exercise: *n* = 11 from six mice). These data suggested that the Vth hyperpolarization induced by exercise training could be primarily determined by Na-PIC rather than Ca-PIC.

Further experiment results showed that exercise training significantly increased the number of APs by 3.8 ± 1.6 (Figure 6H, left panel, unpaired Student’s *t*-test, *p* = 0.025, control: *n* = 10 from five mice; exercise: *n* = 11 from six mice). However, there was no significant difference in AP numbers between control and exercise groups after bath application of nimodipine (Figure 6H, right panel, unpaired Student’s *t*-test, *p* = 0.352, control: *n* = 10 from five mice; exercise: *n* = 11 from six mice), implicating that exercise training might enhance Ca-PC for facilitating repetitive firing.

In summary, the above results confirmed that Na-PIC and Ca-PIC contributed to the repetitive firing of 5-HT neurons in both control and exercise groups and that exercise could enhance Na-PIC for spike initiation and Ca-PIC for repetitive firing regulation.

### Treadmill Exercise Facilitated Dendritic Plasticity

**Figure 7:**
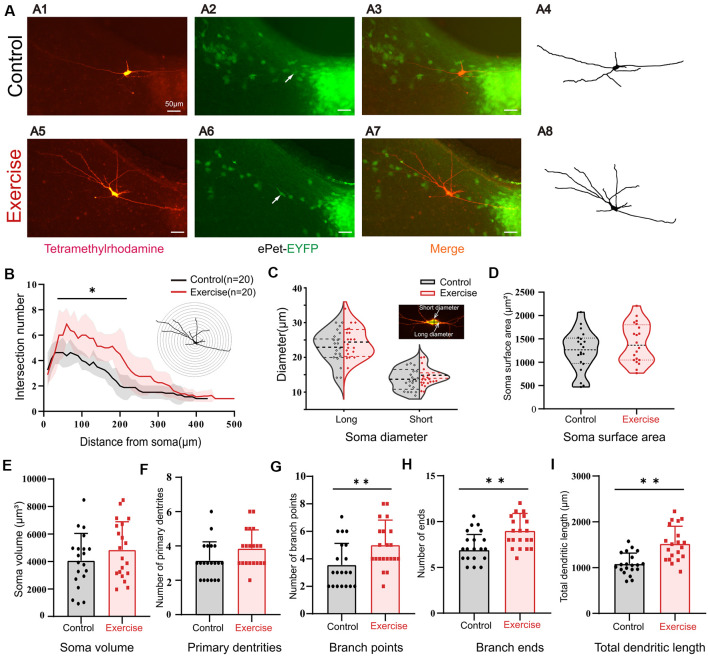
Treadmill exercise facilitated dendritic plasticity. **(A)** Example of exercise training increased the number of dendrites; **(A1)** fluorescence image of staining of tetramethylrhodamine in control group; **(A2)** fluorescence image of EYFP-positive neurons; **(A3)** merged image of **(A1)** and **(A2)**; **(A4)** neuronal morphology diagram reconstructed with Sholl analysis in **(A1)**; **(A5)** fluorescence image of staining of tetramethylrhodamine in exercise group; **(A6)** fluorescence image of EYFP-positive neurons in the exercise group; **(A7)** merged image of **(A5,A6)**; **(A8)** neuronal morphology reconstructed with Sholl analysis in **(A5)**. **(B)** The illustration at the top right is an example of Sholl analysis, in which a series of concentric circles around the soma were created and then counted times the dendrite intersecting the circle (intersections) as well as the length of each dendritic arbor segment within each radius. The number of intersections in the range of 50–200 μm from the soma increased significantly in the exercise group; (*p* < 0.05). **(C)** Most 5-HT cell bodies were fusiform, so we measured the long and short diameters of the cell bodies as shown in the illustration at the top right. Exercise training had little effect on neuron diameters (short diameter: *p* = 0.214, long diameter: *p* = 0.311). There were no significant changes in soma surface area (**D**, *p* = 0.232), soma volume (**E**, *p* = 0.229), and number of primary dendrites (**F**, *p* = 0.054) between two groups. Exercise training significantly increased the number of branch points (**G**, *p* = 0.009), number of ends (**H**, *p* = 0.001), and total dendritic length (**I**, *p* < 0.001). *p < 0.05, **p < 0.01.

It was shown in numerous studies that most of the Ca-PIC originated from dendrites (Jiang et al., 1999). Here, we hypothesized that the effects of exercise on PICs could be generated by synaptic plasticity. In order to study the effects of exercise training on the morphological properties of 5-HT neurons, we added 3% dextran tetramethylrhodamine into the intracellular solution. The Sholl analysis was used to quantify neuronal dendritic properties, such as number of intersections, branch points, terminal points, diameter of soma, and primary segments. The 3-week treadmill exercise significantly increased the dendritic branches, especially within the range of 50–200 μm from the soma (Figure 7B, *p* < 0.05). A typical example is shown in Figure 7A. There was no significant change in the diameter (Figure 7C, short diameter: *p* = 0.214, long diameter: *p* = 0.311), soma surface area (Figure 7D, *p* = 0.232), soma volume (Figure 7E, *p* = 0.229), and number of primary dendrites (Figure 7F, *p* = 0.054) between control and exercise groups. However, exercise training increased the number of branch points (Figure 7G, *p* = 0.009) and ends (Figure 7H, *p* = 0.001) by 1.5 ± 0.5 (from 3.6 ± 1.6 to 5.1 ± 1.8) and 2.1 ± 0.6 (from 6.9 ± 1.7 to 9.0 ± 1.9), respectively. The total dendritic length was significantly increased by 732.0 ± 100.1 μm (from 1,082.1 ± 236.7 to 1,814.1 ± 380.4 μm) in the exercise training group (Figure 7I, *p* < 0.001). These results showed that treadmill exercise facilitated dendritic plasticity, which could in turn contribute to PIC enhancement in the DRN.

## Discussion

Many studies have reported that exercise training could prevent and improve some diseases caused by dysfunction of the serotonergic system (da Costa Daniele et al., 2017; Ji et al., 2017; Ganzer et al., 2018; Kang and So, 2018; Pietrelli et al., 2018), but the cellular and channel mechanisms underlying this effect are not well studied. Using whole-cell patch clamp technology, we discovered that 3-week treadmill exercise enhanced PICs of 5-HT neurons in the DRN, which facilitated the spike initiation and enhanced neuronal excitability. Furthermore, we demonstrated that treadmill exercise enhanced Ca-PIC more than Na-PIC, mainly due to the exercise-facilitated dendritic plasticity.

### 5-HT Neurons in the DRN Were Heterogeneous

The DRN, located in the brainstem, contains the largest population of 5-HT neurons in the brain. These neurons innervate almost the entire brain regions (Ren et al., 2019) and participate in various physiological functions such as emotions, cognitions, sleep–wake regulation, and locomotion control (Abrams et al., 2004; Cabaj et al., 2017; Kawashima, 2018; Bacqué-Cazenave et al., 2020). Accumulating evidence indicates that the functional diversification of 5-HT neurons is not only related to the different regions innervate by 5-HT neurons but also related to the different subtypes of 5-HT neurons (Fernandez et al., 2016; Ren et al., 2019). According to the distribution of 5-HT neurons, DRN can be anatomically divided into ventromedial (vmDR), lateral wings (lwDR), and dorsomedial (dmDR) in the mid-rostrocaudal DRN (–4.5 mm bregma; Hale et al., 2012). Many studies have reported that different subregions of DRN have distinct afferent inputs and efferent projections which lead to different functions of the DRN subregions. Petersen et al. (2020) show that the dorsal (DRd) and lateral (DRl) B7 subregions are primarily responsible for serotonergic innervation of the auditory inferior colliculus (IC) and are related to stressful events (Petersen et al., 2020). Stress is one of the major factors leading to anxiety and depression. In addition, 5-HT neurons in lwDR are predominately recruited with panicogenic stimuli and participate in controlling the expression of panic-associated escape behaviors (Johnson et al., 2005; Vilela-Costa et al., 2019). Recently, the more complex molecular and anatomical diversity of DRN is revealed with single-cell transcriptomics and intersectional genetic tools (Ren et al., 2019; Okaty et al., 2020). In addition to the anatomical diversity of DRN, Calizo et al. (2011) reveal that electrophysiological properties of 5-HT neurons in different subregions of DRN are significantly different. The values of electrophysiological properties of serotonin neurons in DRN vary substantially across neuron subtypes and across species in different studies (Tuckwell, 2013). For example, the resting membrane potential (Em) varies from −57.7 ± 1.1 (DRN, rat; Crunelli et al., 1983) to −63.3 ± 1.9 mV (lw DRN, mouse; Crawford et al., 2010). In our study, the Em of DRN 5-HT neurons in mice was −60.5 ± 3.6 mV in the control group. A large amplitude of AHP is a typical electrophysiological property of serotonin neurons (Vandermaelen and Aghajanian, 1983). The value of the AHP amplitude in mouse is also various in different studies, from 19.9 ± 0.1 (Macri et al., 2006) to 33.7 ± 1.6 mV (Crawford et al., 2010). In this study, the AHP amplitude is 27.4 ± 4.9 mV in the control group. In addition to species and neuronal subtypes, the age of animals is also an important factor affecting membrane properties of 5-HT neurons. Macri et al. (2006) report that serotoninergic neurons undergo significant functional maturation during the first 2 postnatal weeks in mice, and electrophysiological properties change obviously during this period of time. In this study, we demonstrated that the DRN 5-HT neurons displayed diversity in firing pattern and PIC pattern. However, 3-week exercise did not substantially change the proportion of these patterns in firing and PICs.

### Three-Week Exercise Training Facilitated 5-HT Neuron Excitability

Increasing evidences show that exercise can improve depression, anxiety, cognitive disorder, and locomotion impairment by affecting the serotonergic system (Otsuka et al., 2016; Ji et al., 2017; Arnold et al., 2020). Using c-Fos (a marker of acute cellular activation), Otsuka et al. (2016) reported that a 30-min treadmill exercise at low speed (15 m/min) significantly enhanced the activation of 5-HT neurons in the DRN and effectively induced antidepressant/anti-anxiety properties. They further showed that 30 min of highly intensive exercise (25 m/min) had no significant effect on the activation of 5-HT neurons. On the other hand, however, Gomez-Merino et al. (2001) demonstrated that 60 min intensive treadmill running (25 m/min) significantly increased 5-HT release in DRN. These results indicated that the activation of 5-HT neurons was closely related to the intensity and duration of exercise and that the low-intensity exercise seemed to be more effective in activating 5-HT neurons. Similar results were found in the human experiment, where one session of aerobic exercise rather than anaerobic exercise led to a significant effect on serotonin release (Sharifi et al., 2018). In this study, we specifically designed the exercise protocol which contained a flexible moderate intensity protocol, and whole-cell patch clamp recording was performed 24 h following the last training, which allowed 5-HT neurons to adapt to their resting state. Our results showed that 3-week treadmill exercise significantly increased some measures of excitability of 5-HT neurons in the DRN. Interestingly, there was a tendency that the discharge rate of 5-HT neurons in the exercise group was increasingly higher than that in the control group with increment of injected currents (Figure 3H), suggesting that treadmill training might enhance more release of serotonin in mouse during intensive motor behavior such as escape. In addition, Shim et al. (2014) revealed that mouse knee loading upregulated the mRNA level of tph2 in the brainstem, while tail suspension downregulated it. We noted that Veasey et al. (1997) had a different report previously. Their results showed that none of the 5-HT neurons in dorsal raphe demonstrated increased firing during one session of treadmill-induced locomotion in cats. Several possible reasons could account for this different observation, including the difference in animal models, the intensity and duration of exercise protocol, and the preparation for cell recording. Arnold et al. found that 6-week forced exercise increased FosB/ΔFosB, a chronic 5-HT neuron activation marker, expression in 5-HT neurons of rats (Arnold et al., 2020). Alternatively, compared with the sedentary control group, 3-week exercise training could facilitate 5-HT neuron excitability by affecting mood or motivation. Using the optogenetic method to target 5-HT neurons, Correia et al. (2017) revealed that transient 5-HT activation reduced locomotion speed, but repeated daily phasic 5-HT activation for 24 days showed a progressive increase in movement speed, which appeared to arise from the underlying factors that motivate actions. Results from these studies are generally agree with our data that 3-week treadmill exercise significantly increased some measures of excitability of 5-HT neurons in the DRN.

One of the mechanisms responsible for the neural adaptations to exercise training was modulation of ion channels, including acute modulation of channel kinetics and chronic changes in the density, location, or types of ion channels (Gardiner, 2006; Chen et al., 2019). In this study, neuronal properties were measured 24 h after the last exercise training. Thus, changes in the properties should result from modulation of ionic channels for activity adaptation. Our data showed that 3-week exercise training hyperbolized the Vth of the 5-HT neurons with increase in AP amplitude. Both modeling and experiment studies suggested that these changes in Vth and AP amplitude could be produced by upregulating transient sodium channels (Dai et al., 2002; Power et al., 2012). Results from recent studies showed that riluzole, a Na-PIC blocker, significantly depolarized the Vth of 5-HT neurons (Svensson et al., 2017; Cheng et al., 2020), suggesting that persistent sodium channels modulate Vth (Dai et al., 2002; Power et al., 2012). In fact, transient sodium channels and persistent sodium channels are considered to originate from the same ion channels with different kinetics for activation and inactivation (Crill, 1999). Based on these results, we expected that the exercise-induced hyperpolarization of Vth could be modulated by upregulating transient and/or persistent sodium channels of 5-HT neurons in DRN.

### Exercise Training Enhanced PICs and Facilitated Dendritic Plasticity

The most important data reported in this study was that 3-week exercise training enhanced PICs. PICs have been intensely studied and revealed a crucial role for enhancing motoneuron excitability. Activation of PICs leads to self-sustained firing, plateau potentials, and amplification of synaptic inputs, which are mainly mediated by L-type calcium channels (Hounsgaard and Kiehn, 1989; Simon et al., 2003). Additionally, activation of PICs amplifies the synaptic inputs at subthreshold voltages and facilitates spike initiation and repetitive firing, which are primarily mediated by persistent sodium channels (Crill, 1999). In addition to motoneurons, PICs are also found in interneurons (Theiss et al., 2007; Dai et al., 2009b), hippocampal neurons (Muller et al., 2018), Renshaw cells (Boeri et al., 2018), and 5-HT neurons of midbrain and medullar (Ge et al., 2019; Cheng et al., 2020). For the first time, we revealed that exercise facilitated excitability of 5-HT neurons by enhancing Na-PIC and Ca-PIC.

Exercise is one of the essential factors to facilitate the alterations of neuron activity that lead to synaptic plasticity especially during development. This activity-dependent plasticity is retained into adulthood that may promote lifelong brain functions and reduce the risk of brain disorders (Zito and Svoboda, 2002; Perez et al., 2019). Moreover, Serra et al. (2019) revealed that early physical exercise significantly increased dendritic complexity, including increased the number of dendrites, total dendritic length, number of dendritic nodes, and terminal ends of cortical and hippocampal neurons. Further animal studies reported that these effects depended on both animal age and training intensity (de Almeida et al., 2013). Similar findings were discovered in this study that exercise increased 5-HT neuron dendritic complexity in juvenile mice, especially intersections on Sholl rings from 50 to 200 μm, and increased the number of dendrites and total dendritic length in 5-HT neurons (Figure 7). This exercise-induced dendritic plasticity could provide cellular substrate for enhancement expression of L-type calcium channels in 5-HT neurons.

Numerous studies have indicated that L-type calcium channels especially Ca_v_1.2 (α_1C_) and Ca_v_1.3 (α_1D_) are broadly distributed throughout the dendritic trees in brain neurons (Westenbroek et al., 1990; Hell et al., 1993; Obermair et al., 2004; Zhang et al., 2005; Tippens et al., 2008; Leitch et al., 2009). In addition, at the ultrastructural level, Ca_v_1.3 was found in neuronal dendrites of different sizes in the spinal cord of various species (Carlin et al., 2000b; Simon et al., 2003; Zhang et al., 2008). The immunohistochemical and electrophysiological evidence demonstrates that L-type calcium channels increase significantly during development, which parallels the development of behaviors of walking (Jiang et al., 1999). These studies reveal that Ca_v_1.3 (α_1D_) channels are generally located in the soma and dendritic trees of neurons in the central nervous system. Ca-PIC are suggested to be generated mainly within the dendritic trees with L-type calcium channels (especially the Ca_v_1.3 subtype), playing a crucial role in regulating neuronal excitability (Raastad et al., 1998; Carlin et al., 2000a; Li and Bennett, 2003). Results from the above studies generally agree with our data that exercise could induce dendritic plasticity, thus enhancing the nimodipine-sensitive Ca_v_1.3 channels that mediated PIC in 5-HT neurons.

### Early Exercise Benefits to Prevent Mental Disorders

5-HT neurons in the DRN are associated with numerous behaviors ranging from higher cognitive functions to basic physiological regulations (reviewed in Luo et al., 2016). The activity of 5-HT neurons is highly vulnerable to stress; thus, exposure to stressors is a major risk factor for mental disorders. Anxiety and depression are the most common mental disorders in adolescents, whereas the stress often influences adult behavior during development. Early anxiety disorders associated with exposure to stress in childhood may be a marker of dysregulated stress responses (Espejo et al., 2007). Huang et al. (2017) found that postweaning isolation resulted in increased anxiety-like behaviors and had a sex-specific effect on emotional behaviors. Sargin et al. (2016) further revealed that serotonin-producing nerve cells were dramatically less active in early isolated mice and blocking small-conductance Ca^2+^-activated K^+^ (SK3) channels could reverse these anxiety/depressive-like behaviors. In addition, environmental factors such as photoperiod during the developmental period can also affect the 5-HT neuron activity, which in turn can affect mental disorders (Green et al., 2015; Siemann et al., 2019). Therefore, intervention of the serotonin system during development is crucial for preventing mental disorders in adulthood. The beneficial effects of regular exercise on mental health are widely confirmed (Wu et al., 2007; Schuch et al., 2016). McPhie and Rawana reviewed that higher levels of physical activity in mid-adolescence were associated with lower levels of depression during mid-adolescence and slower inclines and declines in depression over time (McPhie and Rawana, 2015). Victorino et al. (2017) observed that physical exercise increases the number of neuronal and non-neuronal cortical cells and hippocampal neuronal cells in adolescent rats. In our study, we revealed that early-age exercise facilitated dendritic plasticity, enhanced PICs, and upregulates the excitability of dorsal raphe serotonin neurons in juvenile mice.

In conclusion, 5-HT neurons in the DRN were heterogeneous and exhibited diverse firing patterns. PICs broadly existed in DRN 5-HT neurons and could influence serotonergic neurotransmission. 3-week treadmill exercise enhanced PICs, facilitated dendritic plasticity, and upregulated excitability of the DRN 5-HT neurons in juvenile mice.

## Data Availability Statement

All datasets presented in this study are included in the article.

## Ethics Statement

The animal study was reviewed and approved by East China Normal University Public Platform for Innovation and the Animal Experiment Ethics Committee (Ethics No. m20190201).

## Author Contributions

YD and RG contributed to conception and design of the study. RG did the experiments and organized the database, performed the statistical analysis, and wrote the first draft of the manuscript. YD and RG modified the main sections of the manuscript. All authors contributed to the article and approved the submitted version.

## Conflict of Interest

The authors declare that the research was conducted in the absence of any commercial or financial relationships that could be construed as a potential conflict of interest.

## Footnotes

^1^http://imagej.net/Sholl_Analysis
